# Development of Gluten-Free Muffins with β-Glucan and Pomegranate Powder Using Response Surface Methodology

**DOI:** 10.3390/foods10112551

**Published:** 2021-10-22

**Authors:** Marcin Andrzej Kurek, Małgorzata Moczkowska-Wyrwisz, Jarosław Wyrwisz, Sabina Karp

**Affiliations:** Department of Technique and Food Product Development, Institute of Human Nutrition Sciences, Warsaw University of Life Sciences, 159c Nowoursynowska, 02-776 Warsaw, Poland; malgorzata_moczkowska@sggw.edu.pl (M.M.-W.); jaroslaw_wyrwisz@sggw.edu.pl (J.W.); sabina_karp@sggw.edu.pl (S.K.)

**Keywords:** response surface methodology, pomegranate, β-glucan, gluten-free

## Abstract

More consumers are being diagnosed with celiac disease or diseases in which wheat products should be avoided. For this reason, it is important to increase the range of gluten-free products available. In this study, it was decided to optimize the technology for the creation of a muffin with β-glucan (BG) and pomegranate (PG), while establishing water share (WT), using the response surface methodology. It was shown that β-glucan and water had the most significant influence on specific volume and moisture (*p* ≤ 0.001). However, the increase of hardness, color, and total phenolic content (TPC) was mainly influenced by the increase of pomegranate content (*p* ≤ 0.01 for harness and color and *p* ≤ 0.001 for TPC). Consumers accepted products high in β-glucan more than high in pomegranate. Optimization ended with a composition that included 1.89% BG, 9.51% PG, and 77.87% WT. There were no significant differences between the model and the experimental sample, apart from higher consumer acceptability.

## 1. Introduction

Celiac disease is more and more frequently being diagnosed throughout the world. This is mainly due to the greater nutritional awareness of doctors, dieticians, and consumers themselves. The biggest problem with celiac disease is that it is not a disease that can be controlled or treated symptomatically. The only known method of fighting celiac disease is to avoid products that may contain gluten. A gluten-free diet is often recommended for people who do not have celiac disease but suffer from other diseases like dermatitis herpetiformis, gluten ataxia, and wheat allergy [1,2,3,4]. For this reason, the gluten-free market is growing more dynamically. A typical diet should contain dietary fiber, unsaturated fatty acids, vitamins, and minerals, as well as bioactive compounds. However, due to technological complexity, gluten-free products often have a relatively low dietary fiber content and, apart from the lack of gluten, do not have a rich nutritional value because the main ingredients of bakery and pastry products are rice flour, tapioca, or corn starch [5].

β-glucan, as a high-molecular-weight polysaccharide, is characterized by the ability to form hydrogels, thus delaying the retrogradation and syneresis processes in bakery products. At the same time, it is classified as a soluble dietary fiber that reduces blood cholesterol levels and lowers glucose and insulin responses, which may lead to the general avoidance of obesity or coronary diseases [6]. Cereal β-glucan can increase the viscosity of aqueous solutions through its high water-binding capacity that leads to gel formation and stabilization of the matrix. Observations made by Karp et al. (2020) [7] indicated that β-glucan could be applied in gluten-free bakery products as a structure-making component.

Pomegranate (*Punica granatum* L.) is a very known source of antioxidants, and each part of the plant contains valuable compounds. Its antitumoral, antidiabetic, antihepatoxic, and antimicrobial properties have been proven [8]. Pomegranate is composed of polyphenols, such as flavonoids (flavonols, flavanols, anthocyanins), condensed tannins (proanthocyanidins), and hydrolysable tannins (ellagitannins and gallotannins), as well as other phytochemicals, such as organic and phenolic acids, sterols, fatty acids, triglycerides, and alkaloids [9]. Pomegranate seeds attract attention due to their high levels of bioactive components and they can be used as an ingredient in muffins.

Confectionery products are consumed in various forms all over the world. However, the quality of cookies depends on many factors, and acceptance is often dictated by the level of consumer habits [10]. For this reason, any attempt to include unusual ingredients in cookies should be characterized by a comprehensive approach to determine the composition and by using statistical tools such as response surface methodology.

In this study, the main purpose was to improve the nutritional value of gluten-free muffins by increasing content of dietary fiber and bioactive ingredients with an antioxidant nature. To the best of our knowledge, up until now, there is no publication in the current literature that has simultaneously aimed to increase both components. For this reason, the study included the addition of β-glucan, pomegranate, and the proportion of water to optimize the technological, physicochemical, and sensory properties.

## 2. Materials and Methods

### 2.1. Material

The research material was based on the gluten-free muffins, the basic product of which was a mixture of rice flour, maize flour, maize, and tapioca starch. The original recipe was based on previous research by Karp et al. (2020) [7]. Rice flour was obtained from Melvit (Olszewo Borki, Poland), and its composition per 100 g of product was as follows: carbohydrate, 78 g; protein, 6 g; fat, 1 g; and fiber, 1.4 g. Corn flour was also obtained from the Melvit company, and its composition per 100 g of product was as follows: carbohydrates, 70 g; protein, 9 g; fat, 4 g; and fiber, 6 g. Corn and tapioca starch were obtained from Vivio (Brzozów, Poland). The thickener used for baking the controls was hydroxypropylmethylcellulose (HPMC) (Sigma Aldrich, Germany) with a viscosity of 4000 cP, 2 wt.% in H_2_O. Barley β-glucan with a high concentration (80–85%) obtained through our own extraction through the Kurek method was used for the research [11]. Pomegranate powder was obtained from seeds (TRS, Hyderabad, India). Sugar, oil, salt, baking powder, and eggs were obtained from the local supermarket.

### 2.2. Preparation of Muffins

The preparation of muffins consisted of combining all the ingredients together in a Kitchen Aid planetary mixer (Kitchen Aid, Benton Harbor, USA) and mixing for 6 min at 110 rpm until a smooth consistency and adequate aeration were obtained. Based on 100 g of the dry base, which was a mixture of flour and starch, the same proportion of ingredients was used in all baked goods in the following manner: sugar (24.6 g), eggs (18.9 g), oil (18.0 g), baking powder (1.1 g), salt (0.1 g), and, in the case of a control sample, HPMC (1.5 g) was used with every 100 g mixture of flour and starch or a mixture of flour, starch, β-glucan, and pomegranate powder. Water comprised 30% to 90% of these blends. All the levels of variables taken into the model are presented in Table 1. After the mixing process, the dough was transferred to aluminum molds and baked for 20 min at 180 °C in a combi steamer (model CPE 110; Küppersbuch, Gelsenkirchen, Germany). After the baking process, the muffins were packed in tight polypropylene bags for future examination.

### 2.3. Physical Properties

#### 2.3.1. Specific Volume and Moisture Content

The specific volume of the muffin was measured by the rapeseed seed replacement method per 1 g of the muffin weight. The weight of the individual cake was recorded, and the results are presented in cm^3^·g^−1^.

Moisture measurement was carried out by weighing the appropriate number of muffins (crumb and crust together) in the form of small particles to facilitate the evaporation of water. The drying process was carried out for 24 h at 105 °C using a convection dryer. The results are presented in % of moisture. Both measurements were carried out with 5 repetitions after 24 h of baking.

#### 2.3.2. Texture, Color, and Porosity

The method of measuring hardness, springiness, and color was based on the measurement modeled by Karp et al. (2016) [12]. Briefly, the muffin crumb was cut into 20 × 20 × 20 mm cubes and was subjected to a double compression test with a Universal Testing Machine Instron 5965 (Instron, Canton, MA, USA). Two successive compressions were performed to 50% of the original height using a 50 mm cylindrical probe. The pre-test speed was 1 mm/s, the test speed was 2 mm·s^−1^, the time was 60 s, and the contact force was 5 g. The force versus time curves were used to determine the hardness and springiness after 24 and 48 h after baking. There were 9 replicates (3 cubes from 3 muffins). 

Color of the crumb was measured with a CR-400 colorimeter (Konica Minolta Inc., Tokyo, Japan) on the L*, a*, and b* CIE Lab scale, where L* stands for (L* = 0 black, L* = 100 white), a* stands for (−a* = greenness; +a* = redness), and b* stands for (−b = blueness *; +b = yellowness *). White reference standard (L* = 98.45, a* = 0.10, b* = 0.13) was used as a calibration. The 9 replicates approach was used. 

Porosity measurement was conducted using computer image analysis using Image-Pro Plus 7.0.1 software (Media Cybernetics, Rockville, MD, USA) based on the Kurek et al. (2017) [13] method, which consisted of taking a photo of the muffin’s cross-section and, after transferring to grayscale, calculating the area covered by the pores and calculating the percentage in relation to the entire surface. The 9 replicates approach was used. 

### 2.4. Chemical Properties

In the field of chemical tests, the total phenolics content (TPC) and antioxidant properties were measured using the 2,2-diphenyl-1-picrylhydrazyl (DPPH) reagent. 

Total phenolic content (TPC) was measured using the modified Folin–Ciocalteu method. Firstly, 1 g of a grounded sample was extracted after 1 h with 10 mL solvent of 80% aqueous methanol with a rotating mixer using the modified method by [14]. After that, the mixture was centrifuged at 2600× *g* for 15 min. TPC assay was conducted by mixing 0.1 mL of supernatant, 0.9 mL distilled water, 1 mL Folin–Ciocalteu reagent (Avantor, Gliwice, Poland) (90% in distilled water), and 2 mL sodium carbonate solution (10% *w*/*v*) (Avantor, Gliwice, Poland). The mixture was kept in the dark at room temperature for 1 h. The absorbance values of the solutions were measured at 765 nm using a spectrophotometer (Shimadzu UV-1800, Shimadzu Inc., Kyoto, Japan) and total phenolic content was expressed as gallic acid equivalent (GAE).

The measurement of DPPH radical scavenging was carried out according to the method indicated by Jan et al. (2018) [15]. The extract from TPC measurement was used for the DPPH measurements. Two milliliters of each sample solution (1.0 mg·mL^−1^) were placed in the tube with 2 mL of 0.1 mM DPPH dissolved in methanol (Avantor, Poland). The mixture was shaken and left for 30 min at room temperature, and the absorbance of the resulting solution was read at 517 nm. The radical scavenging activity was calculated with the following equation (Equation (1)):

(1) $$DPPH\;radical\;scavenging\;activity\;\left(\%\right)=\left[1-\left(\frac{Abs\;of\;extract\;at\;t=30\;min}{Abs\;of\;blank\;sample\;at\;t=0\;min}\right)\times 100\%\right]$$

### 2.5. Consumer Acceptance

The sensory assessment was carried out using a hedonic scale from 1 to 9, where 1 meant “I disliked extremely” and 9 meant “I liked extremely”. The taste, texture, and general acceptability were assessed by a group of 30 semi-trained panelists from the Warsaw University of Life Sciences. All of the panelists were familiar with gluten-free products. The samples were presented to them on the signed plates and the panelists were given tap water to use between the samples.

### 2.6. Statistical Analysis

The statistical method was based on the response surface methodology approach with the simultaneous planning of the experiment according to the central composite design method. Three variables were considered for the research based on the preliminary study to obtain maximum and minimum levels—the content of β-glucan (0.5–3%), the content of pomegranate powder (2–10%), and the addition of water (30–90%). All variables were analyzed using a second-order polynomial equation as follows (Equation (2)):

(2) $$Y_{k}=\beta_{k0}+\overset{n}{\underset{i=1}{\sum }}\beta_{ki}X_{i}+\overset{n}{\underset{i=1}{\sum }}\beta_{kii}X_{i}^{2}+\overset{n-1}{\underset{i=1}{\sum }}\overset{n}{\underset{j=i+1}{\sum }}\beta_{kji}X_{i}X_{j}+\varepsilon$$

where *Y_k_* is the response variable; *X*_i_ and *X_j_* represent the independent variables; *β_ki_*, *β_kii_*, and *β_kj_* represent the linear, quadratic, and interaction regression coefficients; and *ε* refers to the error. The complete design consisted of 15 combinations performed in random order with 5 central points. Each model was analyzed regarding coefficient of determination (R^2^) and lack of fit.

Analysis of variance (ANOVA) was used to observe the effect of individual variables on responses. Additionally, optimization was carried out to determine the optimal values of the input variables, such as specific volume, hardness, TPC, scavenging activity, and overall acceptability. Analysis, optimization, and response surface presentation were conducted with Design Expert 11 Software (Stat-Ease, Minneapolis, MN, USA).

## 3. Results and Discussion

### 3.1. Effect of Pomegranate and β-Glucan on Technological Properties of Muffins

The analysis of variance showed that the lowest value for the specific volume (3.87 cm^3^ g^−1^) was obtained in the Run 4 (lowest β-glucan and water content) and the highest value (4.87 cm^3^ g^−1^) was observed with the highest water content and average β-glucan content of (Table 2) (Run 13). Both β-glucan and water increased the specific volume as linear effects, but vice versa for quadratic effects. The interactions between the factors did not have a statistically significant effect on the specific volume. The pomegranate addition had a negative impact on quadratic terms in reducing the specific volume.

In the case of humidity, the same tests reached the maximum value as in the case of the specific volume. For 0.87% BG (β-glucan), 3.17% PG (pomegranate), 38.79% WT (water) (Run 4), the water content was only 24.27%, while for 1.75% BG, 6.00% PG, 90.00% WT (Run 13), it was 43.56%. Of course, the moisture was significantly influenced by the water refill used to make the batter, while the β-glucan content, in linear terms, was second in order. On the other hand, the quadratic terms of both values reduced the water content in the finished product. The addition of pomegranate did not affect the water content.

Both specific volume and moisture content were dependent on the same factors. They were mainly influenced by the content of β-glucan and the proportion of water. When an insoluble fiber (for example, oat fiber) is added to a gluten-free dough, the volume decreases, but in the case of β-glucan, it usually increases [16,17]. Insoluble fiber increases the frequency of the collapse of the structure, which prevents the maintenance of carbon dioxide bubbles [18]. Meanwhile, in the case of gluten-free baked products, the content of β-glucan may cause a decrease in the specific volume. For the gluten-free dough, β-glucan can act as a thickener and positively stabilize the structure of the baked product [19]. However, interestingly, in increasing the effectiveness and equating to quadratic terms, β-glucan does not give such precise results and may reduce the volume of water. However, this should not be considered individually as the volume was also very dependent on the water content, as the interaction of water and β-glucan slightly increased the value of the specific volume, which is consistent with the studies of Ronda et al. (2015) [20], who showed that β-glucan alone could weaken the structure of the baked product. However, if there is adequate water in the baked product, the volume can increase. The addition of pomegranate did not have a statistically significant effect because, according to Bourekoua et al. [21], the addition of pomegranate should load the structure and negatively affect the specific volume and hydration capacity of the sample. However, when the grant supplement itself was combined with a substance with strong gelling abilities, the effect of the grant supplement itself in our study was statistically insignificant. The specific volume is also influenced by many other parameters, such as the consistency of the cake batter, whipping speed and time, mixture, and baking temperature [22].

### 3.2. Effect of Pomegranate and β-Glucan on Physical Properties of Muffins

The assessment of texture is critical from the consumer’s point of view because it often implies the quality of bakery products to a significant extent [18].

The texture was described using two criteria: hardness and springiness—measured after 24 and 48 h. The increase of hardness after 24 h was most influenced by the content of pomegranate, while the reduction of hardness was influenced by the water content in linear terms. Interestingly, quadratic terms of β-glucan increased hardness, but this was not observed in the case of linear terms either after 24 or 48 h (Table 2 and Table 3). Extending the storage period of muffins from 24 to 48 h increased hardness. However, the factor that had the most significant impact on hardness after 48 h was the interaction between β-glucan and water. In the case of 24 h, this interaction was not observed. This is probably since β-glucan, as a hydrocolloid, absorbs water but releases later on storage phase. It had the lowest water content and the lowest β-glucan content in the sample, and after 48 h, it was 37.14 N. The springiness measured after 24 and 48 h showed a similar tendency to the overall hardness. However, the highest values of springiness were observed in the case of the central sample containing 1.75% BG, 6% PG, and 60% WT (Run 1)—0.87 and 0.74 after 24 and 48 h, respectively. Only the variables in quadratic terms had a significant impact on the reduction of springiness. After 48 h, this group also included β-glucan and water in linear terms and the interaction between them. Importantly, quadratic terms reduced springiness both after 24 and 48 h, but not in nominal terms; these values were not high.

The influence of β-glucan on the hardness of bakery products is unclear. Many studies have indicated that β-glucan weakens the structure of the dough [23,24,25]. Therefore, it has a negative impact on hardness and, thus, the entire value of springiness. However, this argument is mainly significant from the point of view of dough made using gluten flours because hardness is mainly related to the formation of cross-links between partially solubilized starch and gluten proteins [26].

On the other hand, many studies have suggested that it causes a greater viscosity and gel formation and retains water, thus effecting the corresponding viscoelastic values [27,28,29]. This may be caused by a possible inhibition of the amylopectin retrogradation or as a consequence of an increase in the total area of gas cells [19].

Very few studies have used pomegranate in gluten-free applications [10,16,21]. A significant influence on the increase of the hardness value was probably the loading of the structure with a dry powder with a high water absorption capacity. The powder consisted mainly of digestible carbohydrates not observed in the composition or protein, which could contribute in a more comprehensive way to building or disrupting the structure of the dough. In addition, it is worth noting that the pomegranate powder also contains fiber, mainly soluble, which absorbs water and limits its availability to starch and other hydrocolloids, while at the same time building the structure of the dough [30]. The simultaneous increase in hardness leads to a decrease in springiness [10].

The participation of water in shaping hardness and springiness is very crucial because it is the environment where the reactions are taking place, both during the formation of the structure in the mixing process and then during the baking and starch gelatinization process, with the simultaneous denaturation of proteins contained in the dough structure [31]. First of all, the increase in hardness may be due to less swelling of the starch granules during kneading; it then implies a different level of gelatinization of starch during baking and reduces the amylose leaking from the granules. However, the combination of β-glucan and water and its high ability to form gels that retain water may reduce hardness and increase springiness [32].

**Figure 1:**
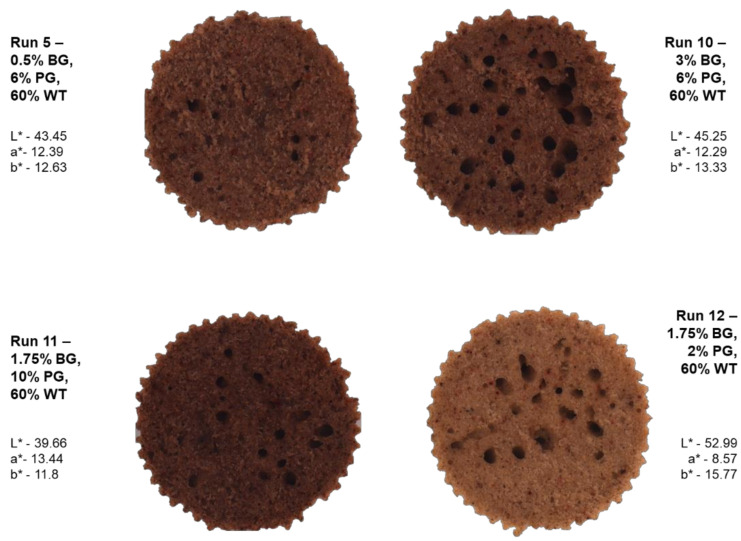
Pictures of cross-sections of muffins with different levels of pomegranate powder, water, and oat β-glucan. BG—β-glucan, PG—pomegranate, WT—water.

The color was described using the CIE L*a*b* color scale, which, in the case of bakery products, is influenced by many attributes, such as ingredients, the combination of temperature and time, and the presence of oven fogging [33]. The significant reduction in L* was influenced linearly by the pomegranate content (*p* ≤ 0.001) (Table 2 and Table 3). In addition, the interaction of β-glucan and water also had a significant effect on the reduction of L*. The increase in parameter a* towards redness was mainly influenced by the content of pomegranate, which increased this parameter in linear terms (*p* ≤ 0.001) (Figure 1). However, an increase in the a* parameter was also observed through the interaction of β-glucan and water. This may be since the sample containing 1.75% BG, 10% PG, and 60% WT (Run 11) was characterized by the highest value of a* (13.44). The decrease in parameter b* was statistically significant due to the addition of pomegranate, expressed in linear terms (*p* ≤ 0.01), and the addition of water because the lowest value was achieved in those samples. For parameter b*, where the water content was quite high, e.g., for the sample with 0.87% BG, 8.83 % PG, and 81.21% WT (Run 14), the result was 11.6. In a statistically less significant way, a decrease in parameter b* was observed in the case of β-glucan interaction with water (*p* ≤ 0.05). Samples with higher scores were closer to shades of yellow. Often, gluten-free bread is characterized by a higher level of the L* parameter than its gluten counterparts, so lowering this value has a positive effect on the perception of color in cookies. In research by Bourekoua et al. (2018), slightly higher levels of the L* parameter were observed as only pomegranate powder was used there. However, garnet showed the most important influence of all color parameters in our research. Β-glucan may slightly increase the color parameters in contact with water, mainly through the dissolution of accompanying components contained in the test preparation. The temperature inside a muffin rarely exceeds 100 °C, so the reasons for the Maillard reactions cannot be found here [34].

Porosity is a feature that shows the ability to support the structure of the dough with trapped bubbles of carbon dioxide and air that have been retained during the kneading process. Generally, the porosity in the samples ranged from 20.76% to 39.32%. Its increase was influenced by the content of β-glucan (*p* ≤ 0.01) and pomegranate (*p* ≤ 0.01), and, at the same time, the water content (*p* ≤ 0.05) decreased porosity in linear terms (Table 2 and Table 3). A negative linear effect on porosity was observed with the water addition, which was consistent with the research of Saeidi et al. [16]. The substantial increase in porosity caused by the addition of β-glucan may explain the fact that the soluble dietary fiber stabilized the structure and, at the same time, affected the content of gas cells in the cake’s structure [35]. The effect of the addition of pomegranate may have been due to the sugar derived from the powder, which acted as a substrate for the fermentation reactions led by yeast. Hence the larger gas cells and their distribution Liu et al. [36] confirmed that an increase in porosity could also mean an increase in volume, which was also the case in our research.

### 3.3. Effect of Pomegranate and β-Glucan on Chemical Properties of Muffins

The motivation for enriching muffins with pomegranate powder was to increase their antioxidant capacity and polyphenol content, which can be measured by DPPH radical scavenging activity and total polyphenol content (TPC) by Folin–Ciocalteu’s reagent. The increase in TPC after adding pomegranate was significant (*p* ≤ 0.001) in linear terms, while β-glucan slightly decreased this content (Table 2 and Table 3). The obtained results were similar to those in Bhol et al. [37] and Bourekoua et al. [21]. However, the high values suggest that the addition of the pomegranate powder increased TPC without much loss during the baking process and exposure to high temperatures. The polyphenol content in the pomegranate itself can be influenced by many factors, such as variety, region, climate, and maturity [6]. The content of polyphenols alone may not drop during the baking process and, at the same time, the rising temperature, but changing the chemical form of phenolic acids may decrease their antioxidant capacity [38]. The fact that β-glucan reduced the content of polyphenols was probably due to the binding of polyphenols in its structure, which prevented proper extraction.

The content of TPC and other antioxidants translated into DPPH radical scavenging capacity. First of all, the addition of pomegranate and β-glucan increased DPPH and radical scavenging activity in general significantly (*p* ≤ 0.001 and *p* ≤ 0.01) (Table 2 and Table 3). The interaction between β-glucan and water also had a significant impact. The lowest inhibition value was 19.2, and it was observed in the case of 1.75% BG, 8.83% PG, and 38.78% WT samples, which proves that it was water that created conditions for the development of features influencing radical scavenging capacity. DPPH radical scavenging capacity values proving the antioxidant capacity resulted mainly from the addition of pomegranate and β-glucan because rice flour has a trace ability to inhibit DPPH radical scavenging capacity—about 8% [1]. The DPPH radical scavenging capacity test measures the ability of an extract to give hydrogen to the DPPH radical scavenging capacity, which causes the color of the DPPH radical scavenging capacity solution to fade. The stronger the observation of fading, the higher the apparent antioxidant activity. DPPH radical scavenging capacity data suggest that the extract is capable of scavenging free radicals, thus preventing the initiation and proliferation of chain reactions mediated by free radicals [37] β-glucan can affect DPPH radical scavenging capacity values, mainly due to the exposure of hydroxyl groups and due to the presence of many anomeric hydrogen atoms, which are mainly broken off by active free radicals [39].

### 3.4. Effect of Pomegranate and β-Glucan on Taste, Texture, and Overall Acceptability Properties of Muffins

Consumer analysis showed that the acceptance of the taste, texture, and overall impression was mainly due to the water content, not the addition of β-glucan and pomegranate. In the case of texture, the increase in acceptance was also associated with the increasing addition of β-glucan (*p* ≤ 0.05), which probably translated into an increase in overall acceptance (Table 2 and Table 3). This is an important observation because muffin consumers pay a lot of attention to humidity, which was also confirmed by the observation that samples with a high addition of water (81–90%) achieved high scores in consumer acceptance tests—above 8.15 on a 9-point scale. Similarly to the study conducted by Bourekoua et al. [16], it has been observed that the addition of pomegranate reduces the overall impression after consumption. This may be due to the slightly sour taste in general, which is dependent on the acids present in the pomegranate. β-glucan is a substance regarding which there is no agreement on the effects of sensory values. However, it has also been shown in other studies that its addition does not adversely affect the sensory characteristics, and even without providing information to the consumer, the difference is imperceptible [3].

### 3.5. Optimization of Muffin Composition

The final stage of the study was to optimize the content of β-glucan, pomegranate, and water. The optimization goal was to maximize specific volume, TPC, radical scavenging capacity, and overall acceptability with a minimum hardness after 48 h. The optimization results indicated the following contents: 1.89% BG, 9.51% PG, and 77.87% WT (Table 4). An experimental trial was carried out, where it turned out that the optimization was performed successfully, and the results were similar to those calculated from the model, except for the acceptability, which turned out to be higher during the experiment approach.

## 4. Conclusions

The present study proved that it is possible to technologically develop a gluten-free muffin composition based on the addition of a substance with high antioxidant potential (pomegranate) and, at the same time, with an increased level of β-glucan, which is also a dietary fiber and technologically functions as a thickener. At the same time, the aspect related to the optimization of the water content had to be taken as one of the main factors comprehensively influencing the dough arrangement. The interaction between β-glucan and water had the most significant number of measured parameters. Interestingly, the final verification of the composition determined by the optimization model showed higher sensory acceptability under experimental conditions than that determined by the model. This is important not only for other researchers who can create a matrix for nutritional or microbiological research, but also for food producers.

## Figures and Tables

**Table 1:** Levels of different variables in coded form for muffin preparation.

Independent Variables	Units	Symbols	Levels				
			−α	−1	0	1	+α
β-glucan	g 100 g^−1^	A	0.5	0.86	1.75	2.63	3.00
Pomegranate	g 100 g^−1^	B	2	3.17	6	8.82	10
Water content	%	C	30	38.78	60	81.21	90

**Table 2:** The central composite rotatable design with process variables and experimental results of responses.

Runs	Variables									Responses									
	β-Glucan (g 100 g^−1^)	Pomegranate (g 100 g^−1^)	Water (%)	Specific Volume (cm^3^ g^−1^)	Moisture (%)	Hardness 24 h (N)	Hardness 48 h (N)	Springiness 24 h (-)	Springiness 48 h (-)	L*	a*	b*	Porosity (%)	TPC (mg GAE g^−1^ d.w.)	Radical Scavenging Activity (%)	Taste	Texture	Overall Acceptability	
1	1.75	6	60	4.51	37.42	14.90	16.54	0.872	0.741	44.78	12.24	12.35	29.21	1.547	21.91	7.63	6.08	7.73	
2	1.75	6	60	4.52	37.24	14.82	16.34	0.861	0.735	44.56	12.18	12.29	31.45	1.536	21.8	7.86	6.48	7.84	
3	1.75	6	60	4.50	37.16	14.79	16.44	0.875	0.738	44.47	12.16	12.27	31.76	1.547	21.76	8.28	6.48	8.15	
4	0.87	3.17	38.78	3.87	24.27	28.83	37.14	0.624	0.521	43.56	12.16	12.34	32.09	0.724	20.42	7.56	5.36	5.97	
5	0.5	6	60	3.92	31.07	21.18	24.22	0.641	0.541	43.45	12.39	12.63	26.88	1.524	19.46	4.64	4.54	4.05	
6	1.75	6	60	4.48	37.05	14.75	16.64	0.851	0.745	44.34	12.12	12.23	28.02	1.554	21.7	5.08	7.96	5.10	
7	1.75	6	30	4.24	28.82	29.50	35.42	0.682	0.574	45.85	12.15	14.06	35.30	1.549	20.27	4.19	3.45	4.49	
8	2.63	8.82	38.78	4.21	32.66	28.32	34.97	0.697	0.598	44.01	11.66	13.5	35.27	2.475	19.46	4.32	3.94	4.70	
9	2.63	3.17	81.21	4.79	37.72	18.04	19.74	0.644	0.602	44.09	12.31	12.48	32.90	0.798	20.67	7.15	5.29	7.29	
10	3	6	60	4.62	37.90	21.79	22.87	0.712	0.694	45.25	12.29	13.33	39.32	0.812	22.23	7.22	5.00	7.39	
11	1.75	10	60	4.42	36.37	25.39	26.27	0.687	0.644	39.66	13.44	11.89	33.28	3.245	32.5	4.56	4.38	4.62	
12	1.75	2	60	4.54	37.46	15.75	17.24	0.623	0.647	52.99	8.57	15.77	20.76	0.524	21.7	5.39	6.74	6.49	
13	1.75	6	90	4.87	43.56	15.54	17.02	0.674	0.654	42.44	12.3	11.47	25.34	1.498	20.89	8.51	6.25	8.15	
14	0.86	8.82	81.21	4.24	35.04	16.76	18.24	0.761	0.705	40.95	11.43	11.6	25.80	2.987	19.2	8.73	5.87	8.23	
15	1.75	6	60	4.48	37.22	14.82	16.17	0.863	0.735	44.54	12.17	12.28	26.69	1.524	21.79	7.11	7.77	8.24	

**Table 3:** Estimated regression coefficients of the fitted second-order polynomial model and their significance.

Parameters	Specific Volume	Moisture	Hardness 24 h	Hardness 48 h	Springiness 24 h	Springiness 48 h	L*	a*	b*	Porosity	TPC	Radical Scavenging Activity (%)	Taste	Texture	Overall Acceptability
Constant	4.5167	37.627	15.096	16.8	0.8483	0.73	44.833	12.45	12.457	29.276	15.08	22.30	6.908	6.807	7.197
β-glucan	0.3500 ***	3.42 *	0.305	−0.675	0.0355	0.077 **	0.9	−0.05	0.35	6.22 **	−0.35 **	1.385 *	1.29	0.233 *	1.666 *
Pomegranate	−0.0600	−0.55	4.820 **	4.515 **	0.0320	−0.0015	−6.665 ***	2.44 ***	−1.940 **	6.26 **	13.60 ***	5.4 ***	−0.42	−1.176	−0.932
Water	0.3150 ***	7.37 ***	−6.980 ***	−9.200 ***	−0.0040	0.04 *	−1.705	0.075	−1.295 *	−4.98 *	−0.025	0.31	2.16 **	1.4 **	1.829 **
β-glucan^2^	−0.2933 **	−4.17 **	5.688 ***	5.810 **	−0.1314 **	−0.101 **	−1.219	0.265 *	0.092	4.19 *	−0.255 *	−2.71	−0.27	−1.666	−0.94
Pomegranate^2^	−0.0833 **	−1.74	4.773 **	4.020 **	−0.1529 **	−0.074 **	0.756	−1.069 ***	0.942	−1.88	0.461 **	3.53	−1.23	−0.876	−1.1
Water^2^	−0.0083	−2.46 *	6.723 **	8.485 ***	−0.1299 *	−0.105 **	−1.424	0.1502	−0.123	1.42	0.105	−2.694	0.15	−1.585 *	−0.34
β-glucan (%) · Pomegranate (%)	−0.0295	2.51	1.30	−0.95	−0.0477	−0.0374	−1.14	0.148	−0.953	−2.71	−0.33	0.443	1.05	1.05	0.16
β-glucan (%) · Water (%)	0.0201	−3.62	7.71	13.22 **	−0.0497	−0.0921 *	−8.08 **	4.132 ***	−2.882 *	10.81 *	−0.05	8.85 **	0.24	−1.24	−1.15
Pomegranate (%) · Water (%)	0.0500	−0.71	0.05 **	4.38 *	0.0722	0.1212 **	−0.52	−0.259	−0.527	3.66	−0.28	1.704	4.24 **	1.33	3.46 **
R^2^	0.9772	0.9612	0.9884	0.9856	0.987	0,9741	0.995	0.992	0.992	0.999	0.9909	0.998	0.995	0.996	0.994
Lack of fit	0.023	0.754	0.125	0.245	0.433	0.022	0.206	0.119	0.14	0.22	0.168	0.147	0.118	0.14	0.265

*—significant at ≤0.05, **—significant at ≤0.01, ***—significant at ≤0.001

**Table 4:** Criteria and outputs of the numerical optimization of the responses with comparison to experimental data.

Optimum Value			
β-glucan (g·100 g^−1^)	1.86		
Pomegranate (g·100 g^−1^)	9.51		
Water (%)	77.87		
Desirability	0.941		
**Variables**	**Target**	**Optimized**	**Experimental**
Specific volume (cm^3^·g^−1^)	Maximum	4.87	4.57
Hardness 48 h (N)	Minimum	16.17	17.12
TPC (mg·g^−1^ dry weight)	Maximum	2.89	2.78
Radical scavenging activity (%)	Maximum	32.52	31.25
Overall acceptability	Maximum	8.23	8.78

## Data Availability

The datasets generated for this study are available on request to the corresponding author.
